# [Corrigendum] Cripto‑1 promotes epithelial‑mesenchymal transition in prostate cancer via Wnt/β‑catenin signaling

**DOI:** 10.3892/or.2024.8734

**Published:** 2024-04-12

**Authors:** Yan Liu, Zhenbang Qin, Kuo Yang, Ranlu Liu, Yong Xu

Oncol Rep 37: 1521–1528, 2017; DOI: 10.3892/or.2017.5378

Following the publication of the above article, an interested reader drew to the authors' attention that the β-actin control blots featured in Figs. 5A and 6A appeared to be strikingly similar. Upon examining their original data, the authors have realized that the β-actin blots for Fig. 5A were inadvertently chosen incorrectly. The corrected version of Fig. 5 is shown opposite. Note that the error made in uploading the incorrect version of this figure did not affect the overall conclusions reported in the paper. All the authors agree with the publication of this corrigendum, and are grateful to the Editor of *Oncology Reports* for allowing them the opportunity to publish this. They also apologize to the readership for any inconvenience caused.

## Figures and Tables

**Figure 5.**
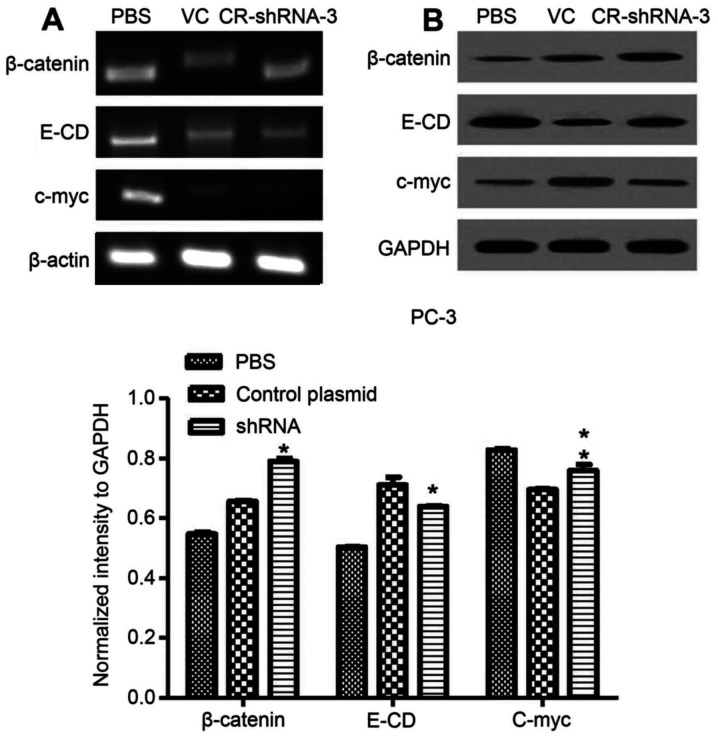
Silencing of CR-1 reverses the EMT phenotype in PC-3 cells. (A) CR-1 silencing of PC-3 cells were EMT-relative at mRNA levels. (β-actin, internal reference). (B) CR-1 silencing PC-3 cells were EMT-relative proteins. (GAPDH, internal reference) (*P<0.01 and **P<0.05).

